# Unraveling the true determinants of citrate accumulation in continuous kidney replacement therapy: the overlooked role of citrate concentration and dilution modality

**DOI:** 10.1186/s13054-025-05816-7

**Published:** 2025-12-29

**Authors:** Minmin Wang, Wenxiong Li

**Affiliations:** 1 Department of Medical Affairs, Vantive Health LLC, Shanghai, China; 2 Department of Surgical Intensive Care Unit, Beijing Chaoyang Hospital, Capital Medical University, Beijing, 100020 China

**Keywords:** Regional citrate anticoagulation, CKRT, Citrate accumulation, Low-concentration citrate solution, Citrate load

## Dear Editor

We read with great interest the recent article by Müller et al. titled “Incidence, severity, and predictors of citrate accumulation during continuous kidney replacement therapy in the critically ill” published in Critical Care [1]. The authors are to be commended for their comprehensive, large-scale retrospective analysis, which provides valuable insights into the incidence and predictors of citrate accumulation during regional citrate anticoagulation (RCA) for continuous kidney replacement therapy (CKRT). Their findings, particularly the robust association between pre-CKRT lactate levels and citrate accumulation, represent a significant contribution towards personalized risk stratification in critically ill patients.

We appreciate the detailed investigation into the association between CKRT modalities and citrate accumulation. The observation of a potentially increased risk with continuous venovenous hemodialysis (CVVHD) compared to continuous venovenous hemodiafiltration (CVVHDF) is intriguing. The authors hypothesize that this might be related to “differences in citrate clearance, as CVVHDF can maintain a consistent ultrafiltration component even when filter patency declines over time” [1]. While this is a plausible consideration, we wish to propose an alternative, and perhaps more fundamental, explanation based on the specific technical configurations and citrate protocols typically associated with the devices used in the study.

In the investigated cohort, CVVHDF was predominantly performed using the Prismaflex system (Baxter, Deerfield, USA), which often employs a pre-dilution mode with low-concentration citrate solution (e.g., 0.5% trisodium citrate, 18 mmol/L citrate) of a balanced pre-dilution replacement fluid to target a blood citrate concentration of 2.5–3.0.5.0 mmol/L [2, 3]. The pre-dilution mode itself is a key factor. By diluting blood before it enters the filter, predilution lowers the ionized calcium (iCa) and hematocrit, thereby allowing effective anticoagulation with a lower absolute citrate dose to achieve the target post-filter iCa (e.g., 0.25–0.5 mmol/L) [4, 5]. Consequently, the systemic citrate load delivered to the patient is reduced.

In contrast, the multiFiltrate device (Fresenius Medical Care, Bad Homburg, Germany) used for CVVHD typically utilizes a high-concentration citrate solution (e.g., 4% trisodium citrate, 136 mmol/L citrate) infused pre-filter without significant predilution [6]. To achieve the same stringent post-filter iCa target (0.25–0.34 mmol/L as mentioned in the supplementary material), this protocol necessitates a relatively higher blood citrate dose (4–6 mmol/L), leading to a greater systemic citrate load [6].

Given that citrate is partially removed by filtration or dialysis with a sieving coefficient approximating 1 [7, 8], the difference in citrate clearance between CVVHDF and CVVHD under stable conditions might be less impactful than the fundamental difference in the prescribed citrate load. The higher incidence of citrate accumulation in the CVVHD group may therefore be primarily driven by the higher initial citrate burden inherent to its typical protocol, rather than a modality-specific superiority in citrate clearance during declining filter function in CVVHDF. This concept of using diluted citrate to enhance safety is gaining traction, as highlighted in recent reviews advocating for protocols that minimize metabolic complications by reducing the citrate load [4, 9].

We kindly suggest that the differential citrate load, dictated by solution concentration and the use of predilution, could be a more direct explanation for the observed association with modality. Disentangling the effect of the modality from the effect of the citrate protocol is challenging but crucial. If the interpretation is solely based on modality, it might inadvertently lead clinicians to prefer one modality over another without addressing the root cause—the citrate prescription itself. A re-analysis stratifying by the citrate dose (mmol/L of blood) or the total citrate load, if data are available, could potentially clarify this relationship further.

Once again, we congratulate the authors on their excellent work, which significantly advances our understanding of citrate accumulation. We believe that considering the role of citrate concentration and infusion strategy will provide an even more nuanced and clinically actionable interpretation of their important findings.

## Data Availability

No datasets were generated or analysed during the current study.

## Abbreviations

RCA: Regional citrate anticoagulation
CKRT: Continuous kidney replacement therapy
CVVHD: Continuous venovenous hemodialysis
CVVHDF: Continuous venovenous hemodiafiltration
